# Editorial: The Dynamic Interplay Between Nutrition, Autophagy and Cell Metabolism

**DOI:** 10.3389/fcell.2021.684049

**Published:** 2021-04-26

**Authors:** Vincenzo Flati, Giovanni Corsetti, Salvatore Papa

**Affiliations:** ^1^Department of Biotechnological and Applied Clinical Sciences, University of L'Aquila, L'Aquila, Italy; ^2^Division of Human Anatomy and Physiopathology, Department of Clinical and Experimental Sciences, University of Brescia, Brescia, Italy; ^3^Cell Signaling and Cancer Laboratory, Faculty of Medicine and Health, Leeds Institute of Medical Research, St. James' University Hospital, University of Leeds, Leeds, United Kingdom

**Keywords:** autophagy, cell metabolism, nutrients and energy, cancer, inflammation

Autophagy (derived from Greek words “*auto”* meaning self and “*phagy”* meaning eating) is a physiological cellular program that removes unnecessary or dysfunctional components in an orderly fashion (Dikic and Elazar, 2018; Platt, 2018). Autophagy allows the degradation and recycling of cellular components. It modulates metabolism of lipids, proteins, or carbohydrates, prevents accumulation of protein aggregates, removes damaged molecules and intracellular organelles and therefore autophagy plays a prominent function in maintaining cell homeostasis, inflammation, neurodegenerative disease and aging (Parenti et al., 2015; Dikic and Elazar, 2018; Platt, 2018) (Figure 1).

**Figure 1:**
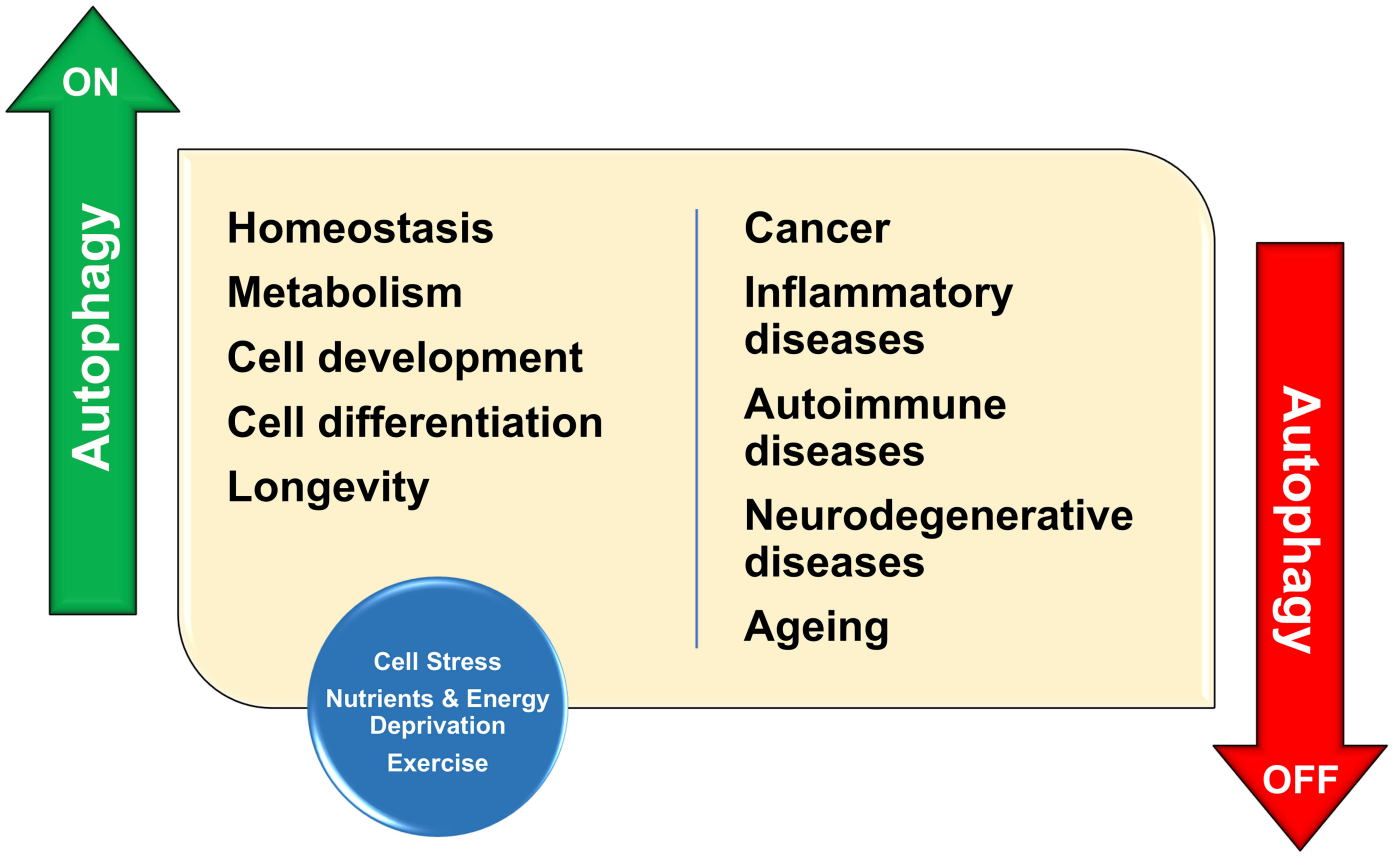
Autophagy has important functions in organelle maintenance, protein turnover, and cellular stress; Therefore, autophagy contributes to the maintenance of cellular homeostasis. As a consequence, any defect in such protective functions would result in the development of many human conditions including cancer, inflammation, and neurodegenerative disease.

The orderly degradation of cytoplasmic content generates by-products that are directly used in many metabolic pathways, therefore autophagy is intimately linked to many metabolic pathways. To emphasize the importance of autophagy in health and disease, we are presenting a Research Topic containing a diversity of Review and Research Articles that shed new light on the molecular mechanisms regulating the interplay between autophagy and cell metabolism in physiological and pathological conditions.

Autophagy has been described in yeast, animals and plants and most of the autophagy related genes (ATG) are conserved among different living organisms (Dikic and Elazar, 2018). In the opening article of this Research Topic, Bu et al., discuss about the role of ATG8 in plant autophagy. Multiple ATG8 orthologs have been identified in plants and the diversity within the ATG8 family may suggest that they are involved in various functions in plants autophagy. More specifically, ATG8 proteins undergo conjugation to phosphatidylethanolamine and regulate membrane elongation during autophagosome biogenesis. This is a process that serves as an important catabolic mechanism in plant growth and development, as well as in plant responses to stress. ATG8s play a role in cargo recognition for selective autophagy by interacting with autophagy receptors/adaptors to target specific substrates for degradation. But the exact roles of ATG8 in plants intracellular trafficking are still poorly characterized. In addition, ATG8 proteins play non-autophagic roles, such as plant senescence control, by interacting with specific proteins. Nevertheless, the broad spectrum of ATG8 functions is yet to be completely clarified.

In another review article focusing on lipid metabolism, Xie et al. discuss the complex interplay between lipid metabolism and autophagy. This interplay is important for the maintenance of cell homeostasis through modulation of cell survival and death. Although lipid metabolism is crucial in the formation of membrane structures that constitute the autophagosome—the double-membrane sequestering vesicles hallmark of autophagy—autophagy *per se* has been found to promote lipid catabolism and lipid peroxidation-induced cell death, such as ferroptosis. A complex connection linking fatty acids, triglyceride, cholesterol, membrane lipids biosynthesis, nutrition status, and autophagy has been also found. The dysfunction of autophagy-dependent lipid catabolism is implicated in several pathologic conditions including steatosis, non-alcoholic fatty liver disease, Parkinson's disease and metabolic syndrome. On this regard, autophagy has been taken in consideration as a target for the treatment of several diseases. Here, the authors suggest that a better understanding of the mechanisms underlining the interplay between autophagy and lipid metabolism would promote potential treatments for lipid metabolism-related disorders. However, many questions remain to be addressed including: how autophagy functions in regulating lipid metabolism in different cells? How autophagy switch from pro-survival to pro-death signal? How selective is autophagy in controlling cell-death?

A review article by Stacchiotti and Corsetti provides an in-depth view of the age-related neurodegeneration, that leads to severe diseases affecting motility, memory, cognitive function, and social life. To date, there is a lack of effective treatments for neurodegeneration and the consequent irreversible neuronal loss. Considering that aberrant autophagy is involved in aging and neurodegeneration, its targeting may represent a possible strategy to fight or prevent age-related neurodegeneration. Many natural compounds such as polyphenols, flavonoids, polyamine, and sugars, limit brain damage *in vitro* and *in vivo* and are modulators of autophagy. Their activity leads to restoration of efficient autophagy thus promoting degradation of misfolded proteins and of dysfunctional mitochondria. Many studies have reported the efficacy of natural compounds in enhancing or restoring autophagy in Alzheimer, Parkinson and Huntington disease preclinical rodent models. However, an enhanced autophagy may be deleterious in forebrain axons, affecting retrograde flux and function. These evidences indicate that in the brain an enhanced autophagy may not be always beneficial. Unfortunately, a limitation to the development of autophagy modulators for therapeutic intervention in humans is that the currently available methods to measure the autophagic flux are not efficient and consequently the generation of natural drug's efficacy in modulating autophagy is complex.

We were also enthusiastic to read a research article by Marzetti et al. discussing physical frailty and sarcopenia (PF&S), a human condition characterized by reduced physical function and low muscle mass in the elderly. In their work, the authors analyzed the relationship between three processes that are thought to be involved in PF&S: systemic inflammation, amino acid dysmetabolism and mitochondrial dysfunction. The studies took advantage of a cohort of old adults recruited in the “BIOmarkers associated with Sarcopenia and Physical frailty in EldeRly pErsons” (BIOSPHERE) study to evaluate inflammatory biomolecules, amino acids and derivatives, and mitochondrial-derived vesicle (MDV) cargo molecules as possible biomarkers for PF&S. The idea behind this primary research was the evaluation of these biomarkers in large cohorts and their changes over time or in response to clinical interventions in order to unveil specific pathogenetic pathways of PF&S and identify new biological targets for drug development. In particular, the retrieval of MDVs in serum of older adults with PF&S has been associated with an innate immune response. According to this theory, MDVs may function as antigen-presenting vesicles carrying harmful material. Similar to damage-associated molecular patterns (DAMPs), released from injured cells, the MDV cargo can trigger caspase-1 activation and the secretion of pro-inflammatory cytokines. Impaired mitochondrial quality control in skeletal myocytes may therefore generate a vicious circle favoring further mitochondrial damage and the propagation of sterile inflammation through DAMPs release. On this regard, the search of circulating mitochondrial DAMPs in the elderly might be exploited for the development of therapeutic interventions for PF&S.

In their mini-review, Kitada et al. discuss about the close relationship existing between nutrients, autophagy and lifespan. In particular the authors focussed their discussion on methionine, an essential amino acid, because of its ability to modulate autophagy through modulation of mTORC1 activity. Recent studies have reported that protein restriction, rather than calorie or dietary restriction, is strongly involved in the lifespan extension and cardiometabolic health. Furthermore, dietary methionine restriction may have a beneficial effect on lifespan extension and metabolic health. Methionine may activate mTORC1 and suppress autophagy. However, it has been reported that mTORC1 may be activated by sensing *S*-adenosyl methionine (SAM) rather than methionine and SAM, rather than methionine, may be the main contributor to the aging process. SAM has been also associated to insulin resistance and truncal adiposity in elderly individuals. Therefore, the upregulation of SAM associated with overfeeding or metabolic dysfunction may be involved in whole-body metabolic impairment. Hence, the suppression of mTORC1, induced by decreasing SAM levels, may represent a therapeutic target for aging, age-related diseases and metabolic impairment.

Last but not least, Thomas et al. report that many clinical trials are evaluating the effectiveness of compounds known to regulate autophagy in patients receiving anti-cancer chemotherapy. In this primary study the authors aimed to assess the effect of amino acid starvation on doxorubicin-treated breast cancer cells by assessing the modulation of autophagy and apoptosis. Indeed, many cancers are known to respond to certain chemotherapeutics or to radiation therapy by increasing autophagic activity. Although the role of autophagy in these circumstances are still debatable, it appears that autophagy acts predominantly as cell survival mediator. In their cell culture-based studies the authors elegantly demonstrated a differential protection of non-cancer cells and increased apoptosis in cancer cells during chemotherapy when these cell lines with high basal autophagy activity were starved of amino acids. Altogether, these results suggest that short-term starvation during doxorubicin chemotherapy may be a possible strategy for adjuvant therapy, especially for the protection of non-cancerous cells.

In conclusion in this Research Topic we have collected a series of review and primary research article describing the close relationships between autophagy and metabolism and we have learnt that many questions are still unanswered and therefore we encourage our colleagues to dedicate quality time onto this fascinating area of research.

## Author Contributions

All authors contributed to the article and approved the submitted version.

## Conflict of Interest

The authors declare that the research was conducted in the absence of any commercial or financial relationships that could be construed as a potential conflict of interest.
